# Sebomic identification of sex- and ethnicity-specific variations in residual skin surface components (RSSC) for bio-monitoring or forensic applications

**DOI:** 10.1186/s12944-018-0844-z

**Published:** 2018-08-21

**Authors:** Satyajit S. Shetage, Matthew J. Traynor, Marc B. Brown, Robert P. Chilcott

**Affiliations:** 1 Research Centre for Topical Drug Delivery and Toxicology, University of Hertfordshire, College Lane Campus, Hatfield, AL10 9AB UK; 2 MedPharm Ltd, 50 Occam Road, Surrey Research Park, Guildford, Surrey GU2 7AB UK

**Keywords:** Skin surface lipids, Ethnicity, Sex, Free fatty acids, High-pressure liquid chromatography-atmospheric pressure chemical ionisation-mass spectrometry, Sebum, Squalene, Sweat, Triglycerides, Wax esters

## Abstract

**Background:**

“Residual skin surface components” (RSSC) is the collective term used for the superficial layer of sebum, residue of sweat, small quantities of intercellular lipids and components of natural moisturising factor present on the skin surface. Potential applications of RSSC include use as a sampling matrix for identifying biomarkers of disease, environmental exposure monitoring, and forensics (retrospective identification of exposure to toxic chemicals). However, it is essential to first define the composition of “normal” RSSC. Therefore, the aim of the current study was to characterise RSSC to determine commonalities and differences in RSSC composition in relation to sex and ethnicity.

**Methods:**

Samples of RSSC were acquired from volunteers using a previously validated method and analysed by high-pressure liquid chromatography–atmospheric pressure chemical ionisation–mass spectrometry (HPLC-APCI-MS). The resulting data underwent sebomic analysis.

**Results:**

The composition and abundance of RSSC components varied according to sex and ethnicity. The normalised abundance of free fatty acids, wax esters, diglycerides and triglycerides was significantly higher in males than females. Ethnicity-specific differences were observed in free fatty acids and a diglyceride.

**Conclusions:**

The HPLC-APCI-MS method developed in this study was successfully used to analyse the normal composition of RSSC. Compositional differences in the RSSC can be attributed to sex and ethnicity and may reflect underlying factors such as diet, hormonal levels and enzyme expression.

## Background

The outermost layer of the skin surface is covered with a mixture of sebum, sweat, corneocyte debris and proteolytic products of filaggrin [1, 2]. This mixture can be readily recovered from the skin surface and is referred to as “residual skin surface components” (RSSC).

Because of its lipophilic nature, continuous contact of RSSC with the immediate environment can lead to absorption of exogenous chemicals [3, 4]. Furthermore, sebaceous glands are believed to have an excretory function and previous studies have shown that the metabolites of ingested chemicals can be eliminated from the body via sebum [5]. Therefore, RSSC offers some potential as a bio-monitoring matrix to assess human exposure to harmful chemicals.

Previous investigations have indicated that skin surface lipid composition varies widely between individuals but appears to be remarkably constant within individuals [6, 7]. In a study by Green et al. [7], the composition of fatty acids of wax esters remained unchanged over a two-month period in an individual, but there was inter-individual variability in wax ester composition [7]. Similar effects in the composition of free fatty acids resulting from hydrolysis of triglycerides were observed by Downing et al. [6]. Such inter-individual variation can be attributed to the subjects’ age and sex [8]. Sex-specific differences in the skin surface lipid composition were also observed by Cotterill et al. [9]; however, Ramasastry et al. [10] did not observe any distinction between the skin surface lipid compositions of males and females. Ethnicity may also affect the composition of skin surface lipids [11]. Therefore, a full understanding of the inherent variability in the composition of “normal” skin surface lipids is an absolute prerequisite for the identification of specific RSSC components with potential use as diagnostic indicators, biomarkers of exposure to environmental pollutants, or in forensic applications.

Various techniques have been used to determine the relative concentrations of different lipid classes of skin surface lipids [9, 10, 12, 13], but information regarding complete lipid profiling identifying individual components is sparse. With advanced analytical techniques, such as gas chromatography-mass spectrometry and liquid chromatography-mass spectrometry, it is now possible to simultaneously obtain detailed information regarding all classes of skin surface lipids [14].

The purpose of this present (human volunteer) study was to investigate the composition of RSSC in different volunteer populations (grouped by sex and ethnicity) in order to establish the “normal” characteristics of RSSC. The study was performed using a standard method for acquiring RSSC samples, with chemical analysis by high-pressure liquid chromatography–atmospheric pressure chemical ionisation–mass spectrometry (HPLC-APCI-MS) followed by sebomic analysis to identify individual components.

## Methods

### Volunteers

Ethical approval to collect RSSC from human volunteers was granted by the School of Pharmacy and Postgraduate Medicine Ethics Committee with Delegated Authority, University of Hertfordshire, Hatfield, UK (ethics approval number: PHAEC/10–25). For the purpose of RSSC collection, adult volunteers (≥18 years old) were recruited at the University of Hertfordshire, Hatfield, UK. All participants were provided with a participant information sheet. All volunteers participating in the study provided written informed consent. Demographic data of all volunteers were obtained using a questionnaire based on the UK “Household Questionnaire Census 2011” [15] and are summarised in Table 1. For the purposes of this study, the “Asian” group comprised individuals from India, Pakistan and Bangladesh. Participants with compromised forehead skin (visual observation) were excluded from the study. For the purpose of confidentiality, all participants were identified with a unique identification number.

**Table 1:** Number of volunteers in different population subgroups. From the total number of volunteers (considered in sex-specific analysis), only those of White, Asian and African ethnicity were considered for ethnic-specific analysis

Sex	Male	Female
	33 (30 ± 12)	58 (26 ± 9)
Ethnicity		
White	8 (39 ± 12)	1 (23)
Asian	11 (29 ± 14)	25 (28 ± 12)
African	5 (25 ± 3)	13 (23 ± 6)

Numbers in brackets represent mean age (± standard deviation) in years

### Sample preparation

Collection of RSSC from the forehead of volunteers was performed for 3 hours using cigarette papers, as previously described [16]. The used papers were immersed in hexane (4 mL) to extract RSSC [17]. The hexane was then evaporated by purging with nitrogen gas to obtain an RSSC residue, which was re-dissolved in 1 mL chloroform:methanol (2:1) using a roller mixer (Stirling mixer, Sandrest Ltd., Sussex, UK) for a minimum period of 2 hours. The resulting samples were analysed by HPLC-APCI-MS. Cigarette papers without RSSC (i.e. not exposed to human skin) were treated in exactly same manner as cigarette papers with RSSC to serve as blank controls. Artificial sebum [18] samples (1 mg mL^− 1^) were prepared in 2:1 chloroform:methanol solvent, which was used as a reference and in the development of the HPLC-APCI-MS method.

### HPLC-APCI-MS

**Fig. 1:**
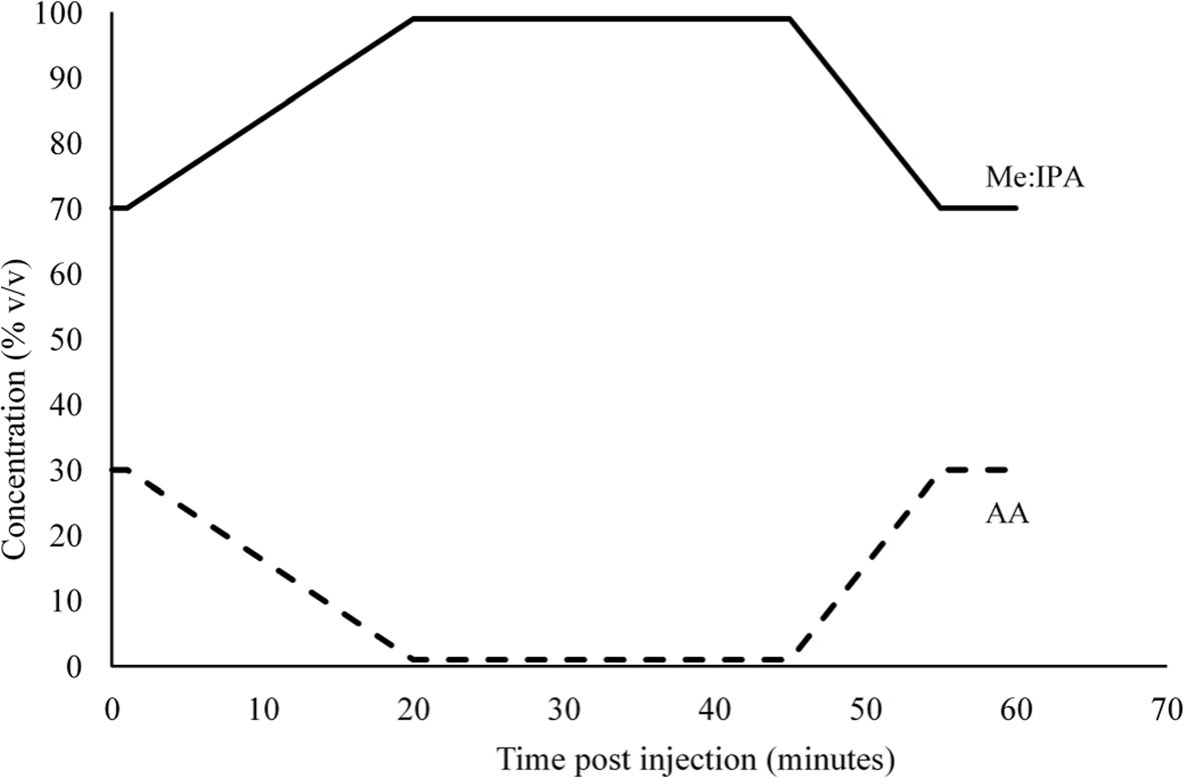
Mobile phase gradient for LC separation of RSSC components. The mobile phase comprised a 95:5 mixture of methanol:isospropyl alcohol (Me:IPA) and an 10 mM aqueous solution of ammonium acetate (AA). The total run time (from injection of sample) was 60 min

HPLC-APCI-MS analysis was performed using a ThermoScientific™ Ultimate 3000 HPLC system, comprising an RS 3000 quaternary pump, Ultimate 3000 RS auto-sampler and a column oven, connected to a ThermoScientific™ MSQ™ single quadrupole mass spectrometer (Thermo Fisher Scientific, Hemel Hempstead, UK). For chromatographic separation, a Zorbax SB C8 column (Agilent Technologies Berkshire, UK), with internal diameter 2.1 mm, length 150 mm and particle size 1.8 μm. was maintained at 60 °C. An aliquot (2 μL) of RSSC sample dissolved in chloroform:methanol (2:1) solvent was injected into the column by the auto-sampler. The mobile phase was drawn from reservoirs containing 95:5 methanol:isopropyl alcohol (A) and 10 mM aqueous ammonium acetate (B). A gradient program (Fig. 1) was employed to achieve chromatographic separation. The mobile phase flow rate was maintained at 0.25 mL min^− 1^ throughout the 60 min run time. The APCI technique was used for ion generation (corona discharge 3 kV, probe temperature 400 °C, cone voltage 50 V). Ions were scanned in the range of 100 to 1000 Da to obtain a total ion scan in both positive and negative ionisation modes.

### Data analysis

The chromatographic data acquired for each sample was converted to “.raw” file format, which was then processed using Progenesis QI software (Nonlinear Dynamics, Newcastle upon Tyne, UK). Compound ions detected in blank solvent (2:1 chloroform:methanol) and control (blank cigarette paper) samples were excluded from the subsequent analysis. Sebomic analysis was performed by peak detection, background subtraction, normalisation and comparison of normalised abundance using the software algorithms provided by the Progenesis QI software: peak detection was performed on the intensity of the compound ions, excluding events with a chromatographic (peak) width of less than 0.1 min. The abundance of compound ions in each sample was normalised against the abundance of compound ions in a reference chromatogram. The final list of compound ion abundances was transformed using an arcsinh function. The transformed data were then used to calculate any significant differences in the normalised abundance between demographic groups. The “frequency of presence” of a compound ion was calculated as the number of volunteers in whose sebum that ion occurred, expressed as a percentage of the total volunteers in the group. A compound ion that was present in more than 75% of the population of a group was considered as a characteristic feature of that group (referred to as a “consistent” compound ion). The normalised abundance of each consistent compound ion was then compared between different demographic groups by either t-test or one-way analysis of variance, using Progenesis QI. A *p*-value of < 0.05 was considered to be significant. Based on their mass to charge ratios (m/z), the components of RSSC were putatively identified based on literature reports [14] and lipid databases (LIPID MAPS).

## Results

### HPLC-APCI-MS

**Fig. 2:**
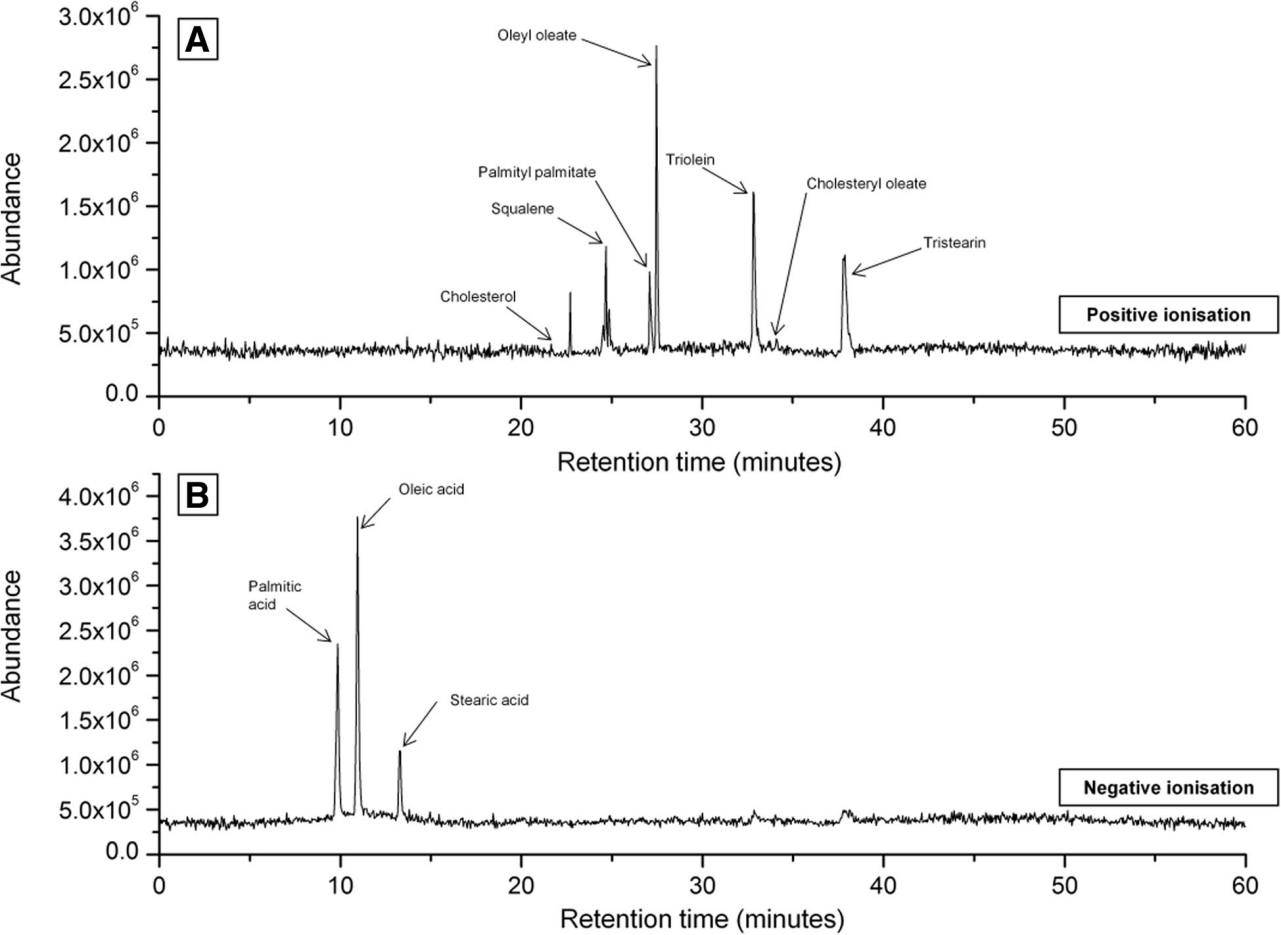
Chromatograms of artificial sebum obtained using HPLC-APCI-MS. Squalene, wax esters, cholesterol and cholesterol ester along with triglycerides were primarily detected in positive ionisation mode (**a**) of mass spectrometry while fatty acids were detected in negative ionisation mode (**b**)

**Fig. 3:**
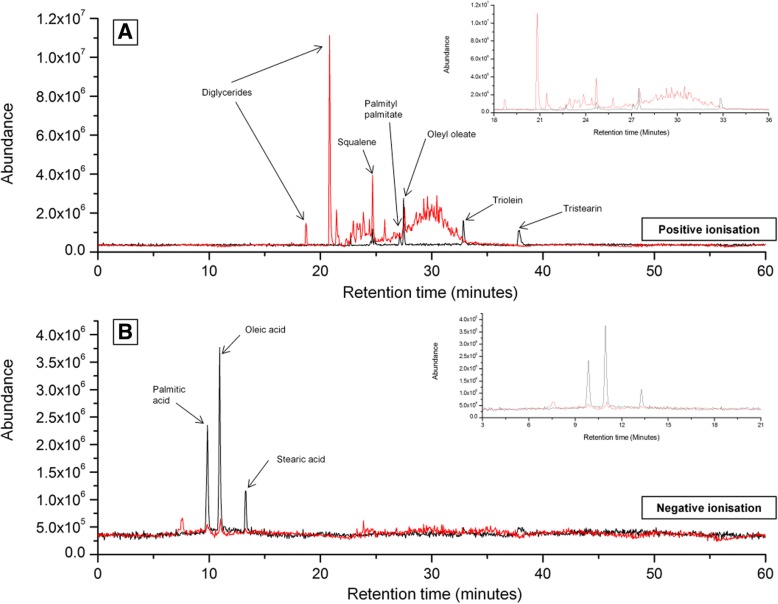
A representative chromatogram of native RSSC (red) superimposed with artificial sebum (black) showing the elution pattern of squalene, wax esters, triglycerides in positive ionisation (**a**) and free fatty acids in negative ionisation (**b**). In addition, two diglycerides were identified in the native RSSC (**a**). The inset figures represent expanded chromatographic regions of interest

Analysis of artificial sebum identified squalene, wax esters (oleyl oleate and palmityl palmitate), cholesterol (CH), cholesterol oleate and triglycerides (triolein and tristearin) in positive ionisation mode and free fatty acid components (palmitic acid, oleic acid and stearic acid) in negative ionisation mode (Fig. 2). These compounds eluted at the same retention time from samples of native RSSC (Fig. 3). Native RSSC chromatograms were generally more complex, indicating the presence of a large number of individual components (Fig. 3), including diglycerides.

### Effect of sex

**Fig. 4:**
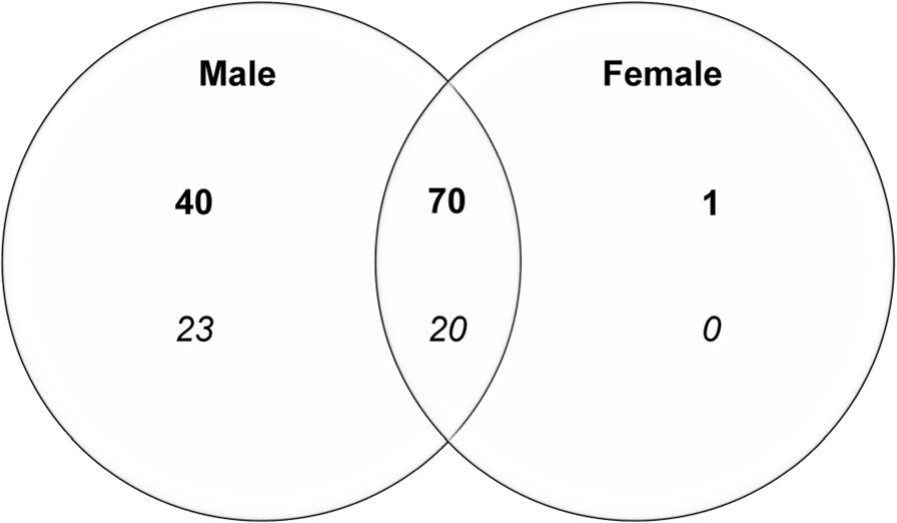
Commonalities and differences in the number of compound ions detected in positive ionisation (bold) and negative ionisation (italic) mode by LC-MS in male (*n* = 33) and female (*n* = 58) RSSC

**Fig. 5:**
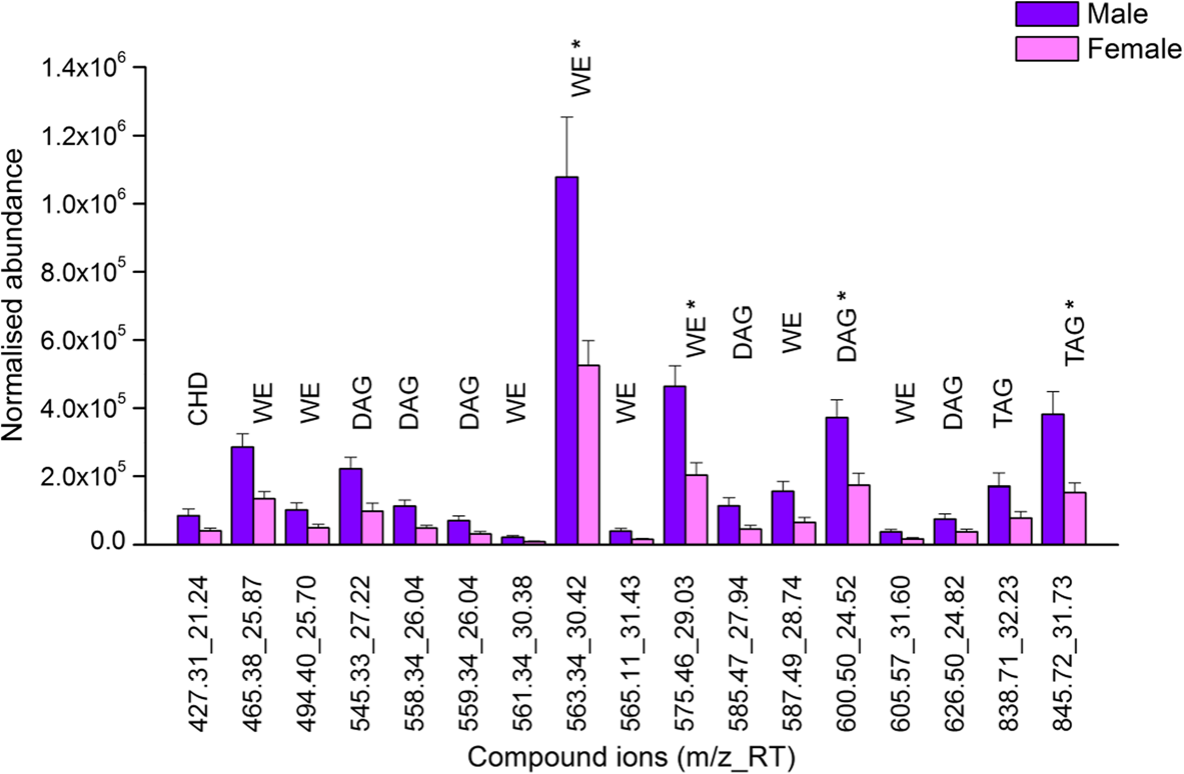
Compound ions with significantly different normalised abundance (mean ± SEM) identified in positive ion chromatogram of male and female RSSC. * indicates compound ions consistently present in both groups. CHD: cholesterol derivative, DAG: diglyceride, TAG: triglyceride, WE: wax ester

A total of 111 RSSC compound ions were detected in positive ion mode, of which 70 were consistently present in both males (*n* = 33) and females (*n* = 58; Fig. 4). The normalised abundance values of four of the 70 compound ions (2 wax esters, 1 diglyceride and 1 triglyceride) were significantly higher (*p* < 0.05) in males than in females (Fig. 5).

Of the remaining 41 positive compound ions, 40 were consistently present only in males and one only in females (Fig. 4). The normalised abundance values of 13 of the 40 male-specific compounds (1 cholesterol derivative, 6 wax esters, 5 diglycerides and 1 triglyceride) were significantly higher (*p* < 0.05) in males than in females (Fig. 5).

**Fig. 6:**
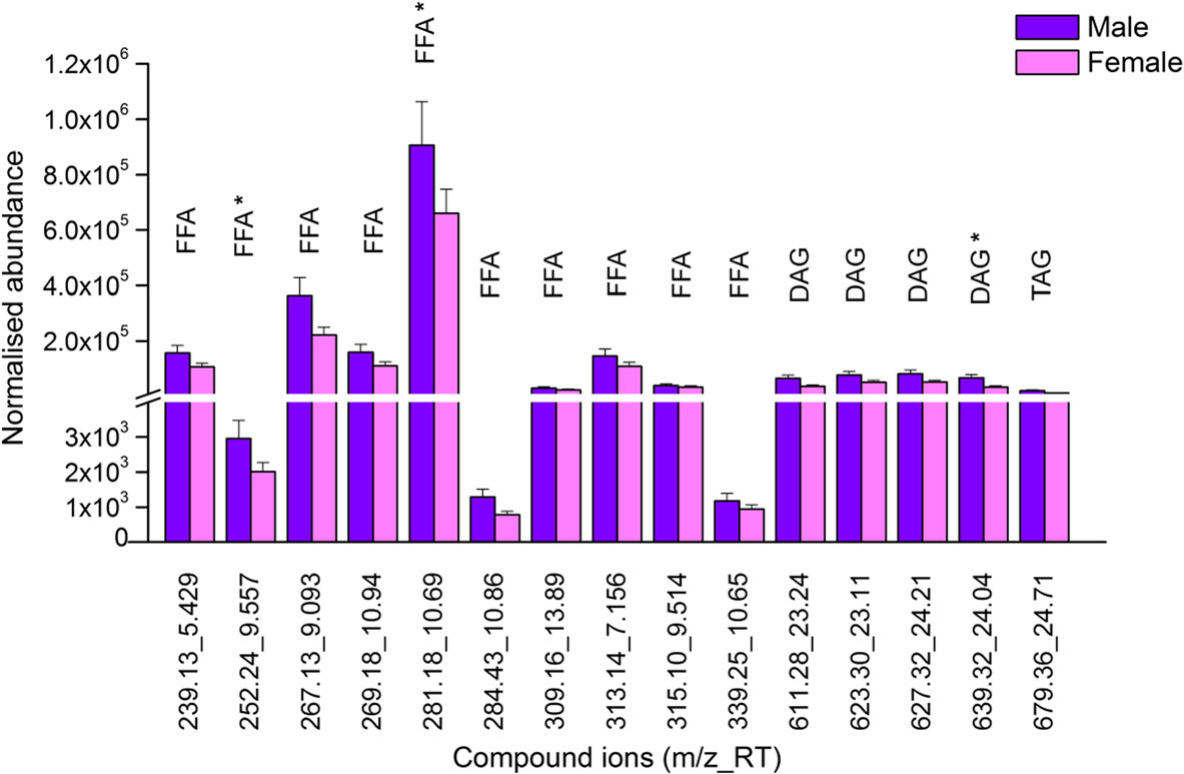
Compound ions with significantly different normalised abundance (mean ± SEM) identified in negative ion chromatogram of male and female RSSC. * indicates compound ions consistently present in both groups. FFA: free fatty acid, DAG: diglyceride, TAG: triglyceride)

In negative ionisation mode, 20 compound ions were consistently present in males and females (Fig. 4); the normalised abundance values of three of these compound ions (2 free fatty acids and 1 diglyceride) were significantly higher (*p* < 0.05) in males than in females (Fig. 6). A further 23 negative compound ions were consistently present in males but not in females; the normalised abundance values of 12 of these 23 compound ions (8 free fatty acids, 3 diglycerides and 1 triglyceride) were significantly higher (*p* < 0.05) in males than in females (Fig. 6).

### Effect of ethnicity

**Fig. 7:**
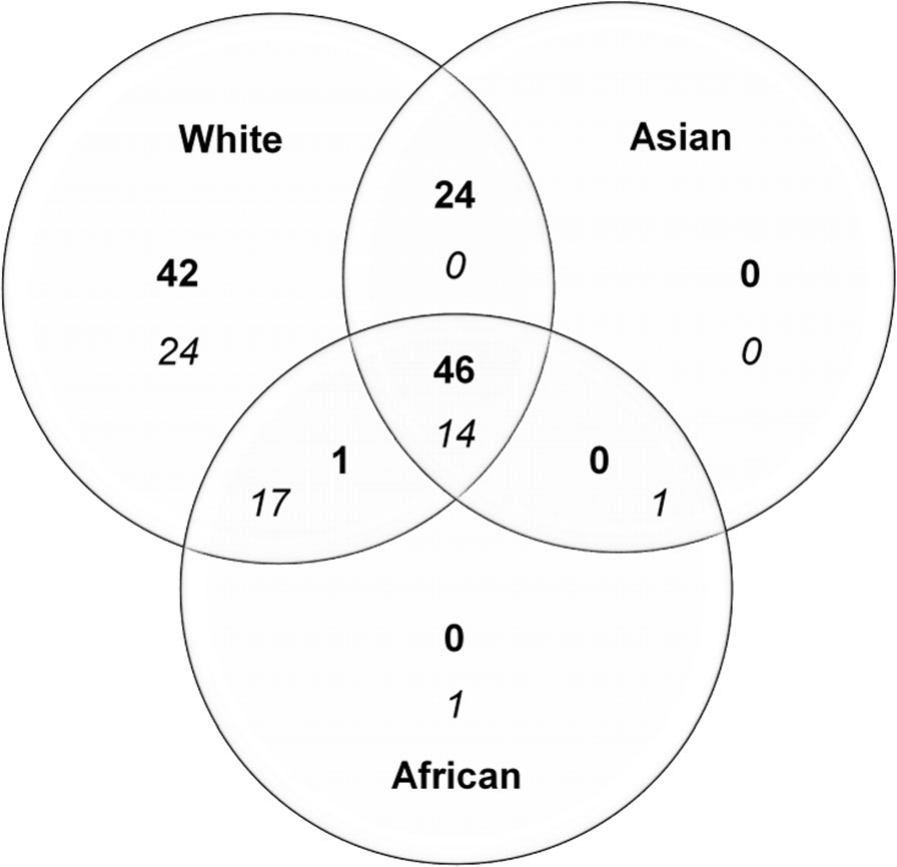
Commonalities and differences in the number of compound ions detected in positive ionisation (bold) and negative ionisation (*italic*) mode by LC-MS in the RSSC of volunteers, grouped according to ethnicity (*n* = 9 White, *n* = 36 Asian and *n* = 18 African)

A total of 113 compound ions were detected in positive ionisation mode from the RSSC of White (*n* = 9), Asian (*n* = 36) and African (*n* = 18) individuals, of which 46 compound ions were consistently present in all ethnic groups (Fig. 7). Of the remaining 67 compound ions, 42 were consistently present only in Whites, one was common to the White and African groups, while a further 24 ions were common to the White and Asian groups. There was no significant difference (*p* > 0.05) in the normalised abundance of any of these 113 compound ions between the three ethnic groups.

**Fig. 8:**
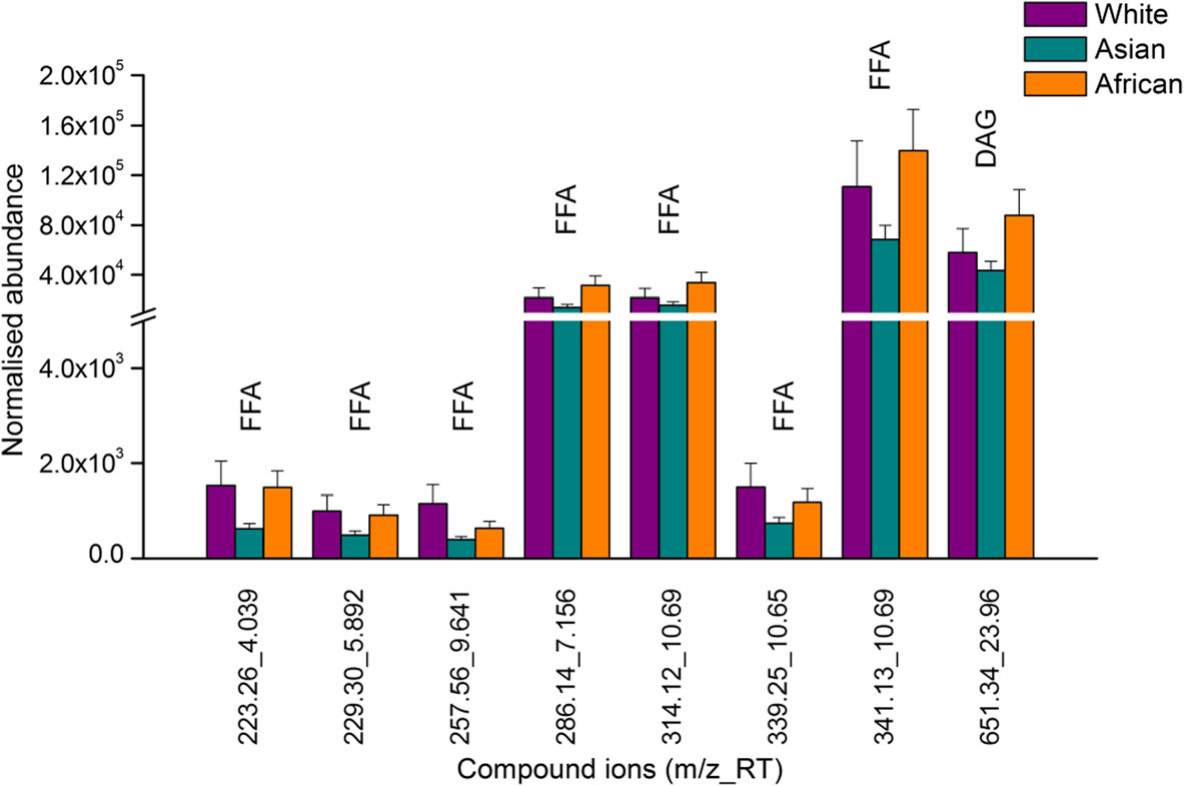
Compound ions consistently present in at least one of the ethnic groups with significantly different normalised abundance (mean ± SEM) identified in negative ion chromatogram of RSSC recovered from White, Asian and African volunteers. FFA: free fatty acid, DAG: diglyceride

In negative ionisation mode, 14 compound ions were common to three ethnic groups (Fig. 7). There was no significant difference (*p* > 0.05) in the normalised abundance of any of these compound ions. A further 24 compound ions were consistently present only in Whites, one only in Africans, one was common to the Asian and African and 17 were common to the White and African ethnic groups (Fig. 7). Of these 43 negative ions, eight compound ions (7 free fatty acids and 1 diglyceride) showed significant differences (*p* < 0.05) in normalised abundance between the three ethnic groups (Fig. 8).

## Discussion

In the current study, we successfully detected and identified components of RSSC that were consistently present and demonstrated significant differences in the abundance of RSSC components tentatively associated with sex and ethnicity. The use of HPLC-APCI-MS enabled the simultaneous analysis of components of major chemical classes of RSSC with minimal sample preparation.

The relative composition of different classes of skin surface lipids has been studied by previous investigators using methods such as thin layer chromatography-densitometry [9, 10, 12]. Such techniques are unable to determine the presence of individual components within each lipid class. Therefore, when the effect of age or sex on sebum composition was investigated previously [13, 19], only minor variations in squalene or fatty acids were observed. However, more advanced chromatographic techniques, coupled with mass spectrometry, allow the detection of individual sebum components. Gas chromatography (GC) is suitable for lipid analysis if derivatisation of lipids is performed prior to GC-MS analysis [20]. However, hydrolysis or decomposition approaches used for derivatisation can modify or ablate characteristic molecular information [14]. Unlike GC-MS, the use of liquid chromatography–mass spectrometry (LC-MS) is not restricted by the volatility of the components, making LC-MS a more appropriate technique to determine compositional changes in RSSC. All representative components of artificial sebum were separated and identified using the analytical method developed in the current study. However, a disadvantage of LC-MS is the inability of certain instruments to identify compounds (readily achievable using GC-MS by comparison of ion fragments to mass spectral databases). In LC-MS analysis, identification of a compound can only be facilitated by the use of sophisticated mass spectrometers (e.g. tandem MS-MS). Since a single quadrupole mass spectrometer was used in the current study, the RSSC compounds detected by LC-MS could not be identified at the molecular level. However, this does not detract from the fact that a number of statistically significant differences were demonstrated in the HPLC-APCI-MS analysis of RSSC; it merely precludes complete identification of the specific compounds.

To investigate commonalities or differences in RSSC composition between subpopulations, a “frequency of presence” was calculated that provided an empirical measure of the incidence of each RSSC component. An arbitrary threshold of 75% was chosen to identify common (> 75%) or absent (< 75%) RSSC components in different sexes or ethnicities. This was consistent with previous biomarker detection studies that reported a range (75–85%) of threshold values [21–23]. Approaches commonly used in “omics”-based analysis were used to analyse a large quantity of sebum lipid profile data; thus, the term “sebomics” is appropriate to describe the work conducted in the present study.

**Fig. 9:**
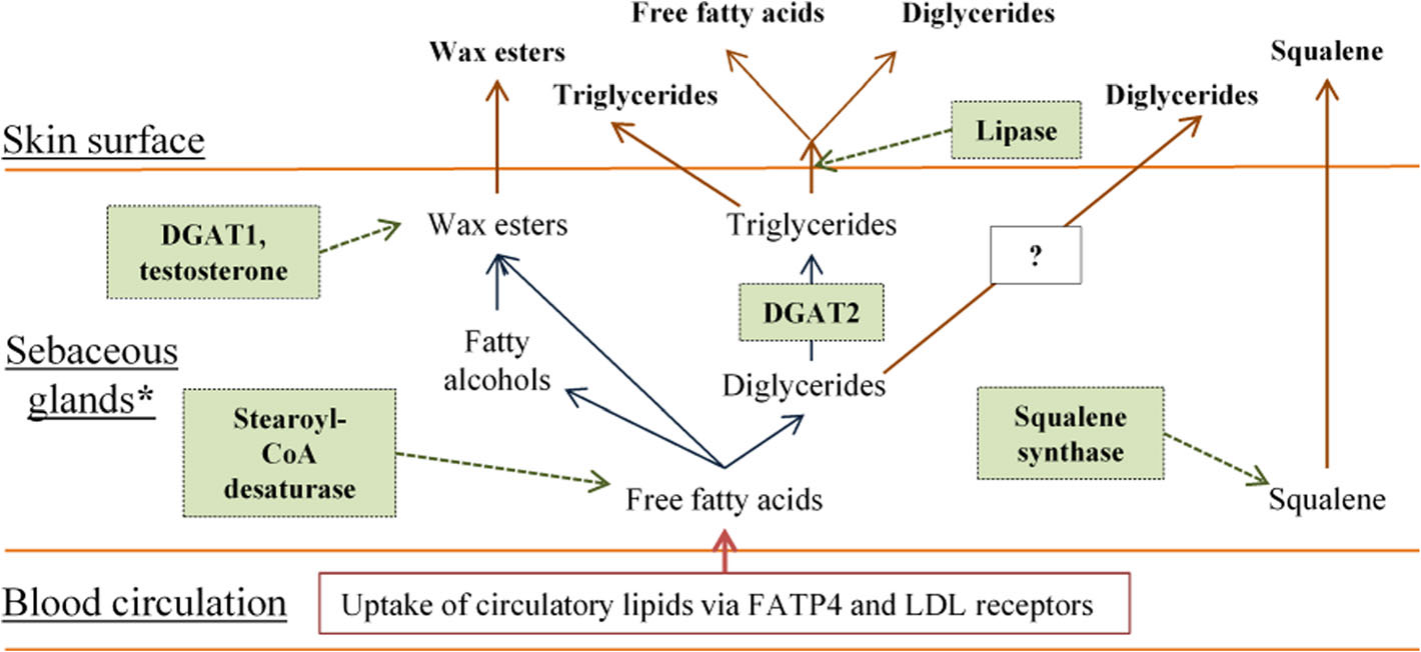
Synthesis of skin surface lipids of sebaceous origin, derived from Smith and Thiboutot [42] and Zouboulis [37] Lipids are synthesised in the sebaceous glands from the substrates, some of which are acquired from the blood circulation. Lipid synthesis in the sebaceous cells (sebocytes) is controlled by various enzymes (indicated by boxes shaded in green). Lipid composition in the glands is different from that on the skin surface as triglycerides are hydrolysed by lipase to liberate free fatty acids and diglycerides on the skin surface. *The examples of receptors present in the sebocytes are listed in Table 2. DGAT- diacylglycerol acyltransferase, FATP- fatty acid transport protein, LDL- low-density lipoprotein

Before discussing the sex-, and ethnic-specific differences observed in the current study, it should be emphasised that the changes in the composition of RSSC may be affected by a complex interplay of several distinct factors (Fig. 9). The composition of RSSC is dominated by sebaceous lipids, whose synthesis is a complex process that produces components unique to sebum [24, 25]. Two receptors expressed in sebaceous glands (fatty acid transport protein and low-density lipoprotein) are involved in the uptake of lipids from the blood circulation. The uptake of circulating lipids is also supported by the observation that, after fasting, the incorporation of free fatty acids into sebum reduces by 20% [26, 27]. Therefore, diet may be an important factor in skin surface lipid composition.

Wax esters are long-chain fatty acids esterified with fatty alcohols [28] and form approximately 26% of skin surface lipids [29]. It has been reported that androgenic stimulation of sebaceous glands can cause an increase in wax ester synthesis [9, 30]. Furthermore, diacylglycerol acyltransferase (DGAT) is an important enzyme for lipid synthesis that is expressed in two forms: DGAT1 and DGAT2 [31]. In particular, DGAT1 is responsible for wax ester synthesis [32]. It has been shown that wax ester production ceases in mice lacking DGAT1 [33]. In sebaceous glands, glycerol is esterified with fatty acids to form diglycerides and eventually triglycerides. Diacylglycerol acyltransferase (DGAT2) is the key enzyme in lipid metabolism that catalyses the terminal step in triglyceride synthesis [32, 34]. Triglycerides, either in the sebaceous duct or on the skin surface, are hydrolysed by lipase to produce diglycerides and free fatty acids [35, 36]. A small proportion of diglycerides, which are not converted to triglycerides, may also appear on the skin surface. However, definitive data are lacking regarding the occurrence of diglycerides in the skin surface lipids [14]. The involvement of various receptors of peptide hormones, neurotransmitters, as well as steroid and thyroid class hormones (Table 2) further enhances the complexity of the lipid synthesis process [37].

**Table 2:** Examples of receptors present in sebocytes and their role in lipogenesis [37]

Receptor	Ligand	Lipogenesis
LXR	22(R)-Hydroxycholesterol	↑
PPAR	Linoleic acid	↑
IGF	IGF-1, insulin	↑
Androgen	Testosterone, DHT	↑
Retinoic acid	Retinoic acid	↓

*DHT* dihydrotestosterone, *LXR* liver X receptor, *PPAR* peroxisome proliferator-activated receptor, *IGF* insulin-like growth factor

Squalene was present in all RSSC samples and no significant difference in normalised abundance was observed between population subgroups (data not shown) [38]. In the presence of squalene oxidocyclase, squalene is converted to squalene-2,3-epoxide, which is further converted to cholesterol [39]. As sebaceous glands have an anaerobic environment, altered activity of oxygen-regulated squalene oxidocyclase can restrict conversion of squalene to cholesterol, leading to a high concentration of squalene in the glands [40, 41]. Furthermore, overexpression of squalene synthase can result in accumulation of squalene in the sebaceous glands [42]. Therefore, it is not surprising that squalene was consistently present. Components of RSSC detected by LC-MS (Table 3) in different population subgroups were categorised according to the chemical class to determine compositional differences. It was observed that the normalised abundance of 10 free fatty acids was significantly lower in females. Females have a lower microbial density on the skin surface than males [43], which may result in reduced lipase activity [9]. The normalised abundance of 10 diglycerides was significantly higher in males; this also seems consistent with the lower microbial lipase activity in females [43]. The frequency of presence and the abundance of wax esters was generally higher in adult males. In this instance, higher (male) testosterone levels may be responsible for this sex-specific effect [44]. The normalised abundance of three triglycerides was also significantly higher in males; this result may be partly attributable to variability in DGAT2 enzyme expression that could be responsible for causing sex-specific differences in triglyceride synthesis [45].

**Table 3:** Summary of RSSC components with significantly different normalised abundance detected by HPLC-APCI-MS in different population subgroups, categorised according to the chemical class

Chemical class	Sex		Ethnicity		
	Male	Female	White	Asian	African
Free fatty acids	10 H	10 L	4 M	4 L	4 H
			3 H	3 L	3 M
Wax esters	8 H	8 L	N/A	N/A	N/A
Diglycerides	10 H	10 L	1 M	1 L	1 H
Triglycerides	3 H	3 L	N/A	N/A	N/A

Each number represents the number of components while the letter indicates whether the abundance was high (H), medium (M) or low (L) in that particular group

Analysis of RSSC collected from adult volunteers of White, Asian and African ethnicity indicated that the normalised abundance of seven free fatty acids and one diglyceride differed significantly between ethnic groups, being generally highest in the African, intermediate in the White and lowest in the Asian ethnic group. A potential source of such interethnic variation may be resident skin flora (which produces lipase), although previous investigations have produced controversial results. Rebora et al. [46] reported higher densities of micro-flora on Black skin than on White skin, whereas Warrier et al. [47] did not observe any difference between the two groups. Therefore, there is insufficient evidence to associate microbial lipase activity with ethnic differences in fatty acids and diglycerides. No significant difference in the abundance of wax esters or triglycerides was detected between the three ethnic groups, which tends to indicate an absence of any of the hormonal or DGAT-mediated effects discussed above.

To date, studies investigating compositional differences in skin surface lipids have mainly been limited to skin conditions such as acne [41, 48] and seborrheic dermatitis [49], with one study identifying a putative association with diabetes [50]. However, altered rates of sebum production in hypothyroidism [51], Turner syndrome [52], Ehlers–Danlos syndrome [53] and rheumatoid arthritis [54] are indicative of modified sebaceous function. Increased sebum secretion is commonly observed in patients with Parkinson’s disease [55, 56]. Therefore, quantification of subtle differences in RSSC composition (“sebomics”) may provide a new strategy for the discovery of diagnostic biomarkers for a range of diseases.

A complete lipid profiling and identification study of all RSSC components should allow the determination of their reciprocal interactions with known metabolic pathways, as discrete changes in the relative abundance of components within a particular chemical class may provide an indication of the underlying mechanism (s) responsible for the variation in RSSC composition associated with disease states, ethnicity, age and sex. Differences in the abundance of lipid classes may arise due to the variability in factors such substrate availability, enzyme expression or hormone levels between different subpopulations, as discussed above. A better understanding of compositional changes in terms of the identity and abundance of lipids present in RSSC could aid in the identification of specific population subgroups and thus have forensic applications, especially as skin surface lipids form the major fraction of “fingerprints” [57]. Moreover, it is not inconceivable that an individual’s RSSC profile may be unique; this aspect is certainly worthy of further work.

## Conclusion

In summary, the results of the present study have indicated that the composition of RSSC could be affected by the subject’s sex and ethnicity in ways that can be determined by the HPLC-APCI-MS method developed in the current study. Factors such as substrate availability and selectivity, enzyme expression and hormonal levels may contribute to the variations in RSSC composition. This proof of concept warrants further lipid identification studies to use RSSC as a bio-monitoring matrix for the detection of markers to be used for disease diagnosis, environmental monitoring and forensic applications.

## Abbreviations

DGAT: Diacylglycerol acyltransferase
GC: Gas chromatography
HPLC-APCI-MS: High-pressure liquid chromatography–atmospheric pressure chemical ionisation–mass spectrometry
LC-MS: Liquid chromatography–mass spectrometry
RSSC: Residual skin surface components
